# Rate of change in longitudinal EMG indicates time course of an individual's neuromuscular adaptation in resistance-based muscle training

**DOI:** 10.3389/fresc.2022.981990

**Published:** 2022-11-07

**Authors:** Filip Stefanovic, Shilpa Ramanarayanan, Nidhi U. Karkera, Radhika Mujumdar, Preethi Sivaswaamy Mohana, David Hostler

**Affiliations:** ^1^ Department of Biomedical Engineering, State University of New York at Buffalo, Buffalo, NY, United States; ^2^ Department of Exercise and Nutrition Sciences, State University of New York at Buffalo, Buffalo, NY, United States

**Keywords:** EMG, fatigue, performance, muscle, quantification

## Abstract

An individual's long-term neuromuscular adaptation can be measured through time-domain analyses of surface electromyograms (EMG) in regular resistance-based training. The perceived changes in recruitment, such as those measured during muscle fatigue, can subsequently prolong the recovery time in rehabilitation applications. Thus, by developing quantifiable methods for measuring neuromuscular adaptation, adjuvant treatments applied during neurorehabilitation can be improved to reduce recovery times and to increase patient quality of care. This study demonstrates a novel time-domain analysis of long-term changes in EMG captured neuromuscular activity that we aim to use to develop a quantified performance metric for muscle-based intervention training and optimization of an individual. We measure EMG of endurance and hypertrophy-based resistance exercises of healthy participants over 100 days to identify trends in long-term neuromuscular adaptation. Particularly, we show that the rate of EMG amplitude increase (motor recruitment) is dependent on the training modality of an individual. Particularly, EMG decreases over time with repetitive training – but the rate of decrease is different in hypertrophy, endurance, and control exercises. We found that the EMG peak contraction decreases across all subjects, on average, by 8.23 dB during hypertrophy exercise and 10.09 dB for endurance exercises over 100 days of training, while control participants showed negligible change. This represents approximately 2 dB difference EMG activity when comparing endurance and hypertrophy exercises, and >8 dB change when comparing to our control cases. As such, we show that the slope of the long-term EMG activity is related to the resistance-based exercise. We believe this can be used to identify person-specific performance metrics, and to create optimized interventions using a measured performance baseline of an individual.

## Introduction

Neuromuscular activity during muscle training has shown to be correlated to contraction response, specifically in the identification of motor recruitment changes and fatigue. Commonly used biopotential measurement systems such as surface electromyograms (EMG) have demonstrated use in these findings, specifically in identifying the patterns of contractile amplitudes and frequencies during a fatiguing exercise (1–6). For example, fatiguing muscles show an increase in EMG contractile amplitude that correlates to the number of recruited motor neurons, as well as a low-frequency shift in the raw EMG signal (7–10). These frequency-based analyses are a popular strategy to measure the presence of fatigue and adaptation in short-term applications such as on a set-to-set basis (2, 11, 12). However, they have not yet been used in measuring the long-term effects from neuroadaptation (i.e., anti-fatiguing response) due to prolonged regular training. Thus, it is currently unknown how these behaviors change over the long-term, and how specific muscle training over weeks or months impacts these trends.

It is believed that these observations can have an impact on various neuromuscular training applications such as athletic training optimization as well as neurorehabilitation therapies (13–18). It is traditionally observed that the outcomes of these types of training are strongly correlated with the activity time (19–26). For example, limitations in muscle performance – such as neuromuscular fatigue – can significantly impact the quality of outcome in muscular training. However, current methods that identify these effects are largely subjective and driven by qualitative observations of a participant's performance (27, 28). As such, enhanced quantitative methods must be applied to improve performance during muscular training for optimized training strategies.

Currently, it is understood that muscle fatigue can be measured in several ways. An increase in the Root Mean Square (RMS) of the EMG during a fatiguing task is directly related to the recruitment of additional motor units while performing the task (29). Similarly, the time-domain increase in EMG peak amplitude during submaximal fatiguing contractions has similar increasing trends during additional motor unit recruitment up to a maximal fatiguing state (30). Once the motor units reach recruitment limits the muscle experiences a loss in tension, and the force output of the muscle decreases. This phenomenon is then measured by a subsequent decrease in the EMG amplitude during exercise. So, if the EMG RMS and peak-contractile amplitudes increase with motor recruitment, and then decrease following motor unit recruitment limits, it is hypothesized that a quantitative metric can be developed to ascertain an estimate of the rate of motor unit recruitment, along with changes in performance over time.

In this study, we will demonstrate that with regular muscle training the rates of motor recruitment change over time. Specifically, we hypothesize that the rate of EMG peak progression will decrease thereby indicating adaptation to a specific training strategy. We accomplish this through endurance-based and hypertrophy-based resistance exercises using healthy participant (31). We will demonstrate that time-based EMG activity can capture short-term perceived fatigue changes (during each set in an exercise), as well as long-term perceived fatigue or anti-fatiguing changes to muscles over 100 days of regular exercise. These rates of adaptation will be unique to each participant but will also demonstrate, with caution, a generalized behavior based on the exercise regimen. As such we postulate that the rates of change of these EMG slopes will be exercise dependent (e.g., heavier weight vs. more repetitions).

Ultimately, these observations lead to a novel hypothesis that suggests muscular contractions in a participant-normalized EMG (dB/day and *Δ*dB) can be used to measure long-term changes in their muscle performance including the identification of rates of fatigue, performance improvement, and anti-fatiguing response. The resultant trends may have different rates based on the type of exercise, but also that those who do not regularly train (control participants) do not experience the same kind of adaptation. The work we present herein is part of a larger study toward building optimized person-specific strategies for neurorehabilitation therapies, and athletic performance.

## Methods

### Participant recruitment

Data collection for this study was approved by the Institutional Review Board (IRB), University at Buffalo. Informed consent was obtained from all the participants and the data obtained were de-identified. Participant recruitment was performed based on the following inclusion and exclusion criteria;

Inclusion criteria:

• The participants must be within the age group 18–30 years.
• Participants must have a BMI in the range of 18.5–24.9.
• Participants must self-report as being healthy and able to lift low to moderate weights.

Exclusion criteria:

• Participants who have reservations against lifting weight within a comfortable limit of their own threshold (based on a 1-repetition benchmark).
• Participants who are undergoing or have previously undergone treatment for cardiovascular, muscular, or other health concerns
• Participants who refuse to provide informed consent.
• Individuals actively participating in fitness programs or currently following an exercise routine at a gym, including weightlifting.
• Pregnant women.

A total of 11 participants were recruited, 4 performed the hypertrophy exercise, 4 performed the endurance exercise, 3 were assigned to the control group (of which 2 performed modified hypertrophy exercises, and 1 performed a modified endurance exercise). The selected test participants were between 23 and 26 years of age, and included males and females. We would like to identify that due to the length of the study, particularly in requiring inactive individuals to participate in regular exercise for over 3 months, it proved difficult to recruit a larger cohort.

### Device

**Figure 1:**
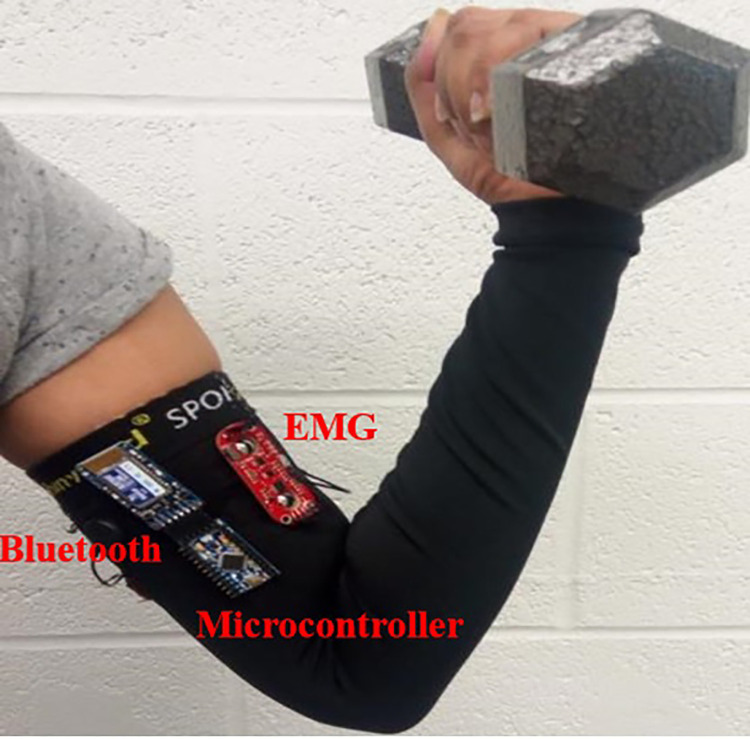
Data acquisition device schematic during experimentation. An Arduino Pro mini was connected to the EMG sensor, and a Bluetooth device for wireless data transmission. The Arduino and EMG boards were separately powered by 9 V batteries.

For this study, we developed a wearable EMG sensory device that collects EMG data, and wirelessly transmits them to a PC for storage and analysis. Figure 1 shows the electronics used for acquiring these muscle activity data. The device consists of a microcontroller (Arduino Pro Mini 328, Arduino, Italy), three rechargeable 9 V batteries, a Bluetooth shield (Bluefruit EZ-Link Breakout, Adafruit, United States) and an EMG sensor (Muscle sensor v3, Advancer Technologies LLC, USA). The microcontroller and the EMG sensor have built in features for optional signal pre-processing. The microcontroller is powered externally by a 9 V rechargeable battery and supplies the Bluetooth shield. The EMG sensor is powered externally by two 9 V batteries (±9 V required supply). We used standard wet electrodes for this study that snap into the Muscle Sensor v3. The same device is used for both endurance and hypertrophy-based EMG studies.

### Experiments

For this study we included two exercise groups of interest – hypertrophy and endurance. The hypertrophy group included an intermediate level of repetitions (10–12) using a weight that is approximately 70%–75% of their 1 repetition strength benchmark (32). The endurance group was required to perform a higher level of repetitions (∼20) with a lower weight. Our earlier proof of concept study examined repetitions until failure with promising results, so we selected a high repetition count for our endurance study (33). On the first day of testing all participants were required to perform their maximum weighted 1 repetition bicep curl with qualitatively observed correct form. This was performed as follows: (1) The participant stands with feet shoulder width apart, holding the dumbbells with both hands in a relaxed position (down at the sides); (2) The participant raises the weights towards the shoulders with palms facing upwards and elbows tucked closely near the ribs/hips; (3) The participants shoulders should be relaxed and their back/posture should not change during the lifting motion; (4) The weight is returned to the starting position in a relatively slow and controlled fashion while exhaling.

After completing this each individual was assigned dumbbell weights (one dumbbell per arm) based on their own ability. All participants appeared to be of similar capabilities with similar 1-repetition maximum weight – recall, these are all participants who have not regularly weight trained. As a result, all hypertrophy study individuals were assigned 10 lbs, while the endurance study participants were assigned 5 lbs. One control participant was assigned a 20 lb weight for hypertrophy exercises, while the other two control participants were also assigned 5 lbs. For all experiments, each participant was required to perform a standing bicep curl with their assigned weight – this is a contraction of the bicep toward their face, and then a controlled return back to the starting position. We selected the bicep curl exercise due to the ease of isolating a large primary mover in the arm that is easily accessible for EMG measurements. Additionally, bicep curls are also easy to control, resulting in fewer motion artifacts during data recording. The Hypertrophy and Endurance groups performed their exercises twice per week for the duration of the study, while the control participants performed their exercises once per month.

During the recruitment process we instructed participants that they should go about their regular daily routines and not introduce any new activities. Since the exclusion criteria specified that participants should not be engaged in any fitness activities, the likelihood of variability in experimentation due to other activities is minimal. However, we still acknowledge that variability will still be present in measurements due to daily variation in physiological function, disposition, or other uncontrollable phenomena.

#### Hypertrophy based exercise

Hypertrophy experiments were conducted over a period of up to 100 days. In this exercise group, participants performed 4 sets of bicep curls, 12 repetitions each with a dumbbell. Between each set, the participant was given 1 min to rest. The exercise was performed at a rate of ∼0.4 Hz, which was controlled using a metronome. The hypertrophy participants performed this exercise twice a week, every week. Data were obtained over a period of approximately 100 days.

#### Endurance exercise routine

A bicep curl exercise using a dumbbell was chosen to isolate biceps muscle activity for endurance-based exercise. Every participant was required to perform 20 ± 2 repetitions of 5 sets per exercise schedule with a dumbbell. We used a metronome set at ∼0.4 Hz to achieve a standardized repetition rate. They were then allowed take a break for approximately 1 min, and continued on to their next set. The endurance participants performed this exercise twice a week, every week. Data were obtained over a period of approximately 100 days.

#### Control exercise routine

Control exercises are meant to be identical to the mentioned exercises, however performed less often. For example, two participants performed the hypertrophy control exercise once a month, while one participant performed the endurance control exercise once a month. These exercises have the same number of repetitions and sets as their matched group.

### Data acquisition

The Muscle Sensor v3 acquires EMG data using bipolar electrodes positioned at the center and end of the bicep muscle body as well as a reference electrode at the elbow joint. The placement of the poles varies based on participant morphometry but is typically around 44 mm (<2 inches) as per the specifications of the system. The electrodes are placed using the participant's anatomical landmarks, in an attempt to minimize variability in electrode placement. For example, the participant bends the elbow to a 90° position, and the electrodes are placed about the point which is 1/3 of the distance from the fossa cubit along the medial acromion-fossa cubit line.

During exercise, the EMG sensor collected the raw and filtered data of the muscle activity that were then sent to the Arduino Pro Mini. These data pins include a 10-bit analog to digital converter which returns integer values from 0 to 1023. The microcontroller converts these analog values from a recorded voltage of 0–5 V. We also included a Bluetooth module to transmit data wirelessly to a laptop (∼10 m range). Then, CoolTerm was used as a serial port terminal for data transfer to collect the data from the Bluetooth shield. CoolTerm is an open-source software that allows the exchange of text/ascii and other data between connected serial ports.

**Figure 2:**
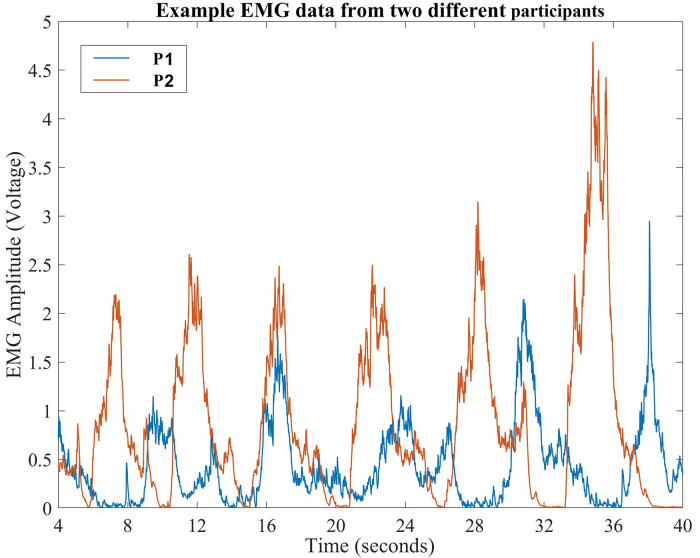
Typical data recorded in our study showing variability in EMG amplitudes during muscle contraction of during exercise. Peak amplitude continuously increases with each contraction.

The Arduino was programmed to begin data collection as soon as the device was powered on. By enabling the serial connection, the data were transmitted *via* Bluetooth to the laptop/computer. Then, the data were stored in row formatted text files with voltage values between 0 and 5 V (∼10 mV resolution), with time stamps (see Figure 2). We loaded these files into MATLAB for analysis upon which all spurious data from the start-up and power-off phase of data collection were trimmed during data post processing. The sampling rate of the EMG was the standard Arduino rate for analog pins (9,600 Hz).

### Data processing

To trim these data, we identify the start and stop times of the exercise by visually inspecting the cyclical contractions of the bicep curls (e.g., first peak in cycle to last peak in cycle – if there are 10 repetitions in an exercise there will be 10 periodic peaks). Once these times are identified visually, we remove data preceding the first time point, and as well as the data succeeding the last time point.

**Figure 3:**
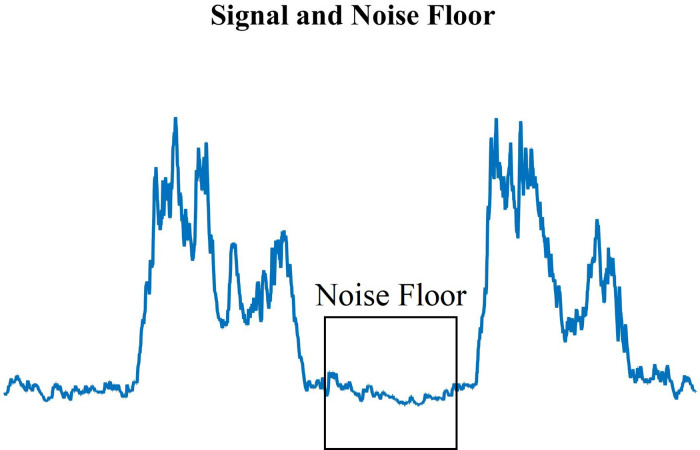
Standard EMG muscle contraction peaks and the measured noise floor (highlighted area) during muscle relaxation. The noise floor was determined manually for each recording.

For each measurement, a zero-mean rectified EMG was created by subtracting the mean voltage of the signal noise floor (Figure 3) – including DC offset – then rectifying. The rectified EMG is then low-pass filtered to 40 Hz since the dominant energy of interest in this study is in the low-frequency bands (34, 35) and we are interested in the behavior of the contraction waveform, rather than the switching properties in the muscle (36). Since the bandwidth of motion for the exercise is low (∼0.4 Hz) this is also well within the envelope of the rectified EMG signal, and appropriate for study (35).

The amplitudes, in volts, of the digitally sampled raw and filtered data were plotted with respect to time (seconds) to provide information regarding how the EMG amplitudes change with muscle contraction in an ongoing exercise. These data allowed us to track EMG peak amplitudes and calculate the change in peaks over time. Previous studies have indicated that these peaks increase with fatigue and motor unit recruitment (37–39), so we wish to measure the rate at which this occurs in either volts per second, or dB (V/V) per second. The times were obtained from the device by dividing the data index (which corresponds to the number of samples) in the time stamp.

The EMG relative peak amplitude in decibels is used to describe the relative change in the data with respect to the sensor's noise floor. As described above, we hypothesize that this relationship can indicate not only how the EMG signal changes with respect to repetitions, but also to measure its long-term performance change with respect to previous exercise dates. As such, we argue that it is a compelling relative metric to measure or identify properties in the muscle in response to fatigue, or anti-fatiguing, due to repetitive exercise. Thus, the change in the ratio with respect to time (i.e., the slope of EMG vs. time) can show changes to motor unit recruitment in the muscle over time (6).

The EMG ratio can be calculated by using the ratio of the signal and power of a reference signal in decibels. For this study we define the reference voltage as the noise floor (i.e., measurement when the muscle is not flexing) and is expressed as:

(1) $$\mathrm{EMG}\;\left(\mathrm{dB}\right)=20\;\log_{10}\left(\frac{\mathrm{Signal}\;\mathrm{Voltage}}{\mathrm{Reference}\;\mathrm{Voltage}}\right)$$

where, the signal voltage is the muscle contraction measured at the skin surface using the EMG sensor in volts, and the reference voltage is the average noise floor as measured by the sensor for the recording. Thus, data are normalized in each set recording – the reference voltage can be different in each recording. So, the EMG ratio (peak to noise floor) in each recording measures the progression of contraction amplitude during exercise. For example, if the noise floors are different across days this does not affect the results since the ratio records the peak to noise floor relationship, allowing us to investigate how the EMG amplitude grows during each set of exercise. Therefore, this allows us to compare EMG amplitude increases (i.e., fatigue) across each day. To do so, we take the peaks of the EMG(dB) and average across the set. Then, to compare across multiple days, we will take the EMG peak ratio on day 1 (starting day) as the base-line. So, we will subtract the EMG peak ratio value from all days as a way to normalize the EMG peak relationship as follows:

(2) $$\mathrm{EM}G_{n}\left(i\right)={\mathrm{EMG}}_{\mathrm{avg}}^{\;\mathrm{peaks}}\left(i\right)-{\mathrm{EMG}}_{\mathrm{avg}}^{\;\mathrm{peaks}}\left(\mathrm{baseline}\right)$$

where *i* is the *i*^th^ day of recording, and ${\mathrm{EMG}}_{\mathrm{avg}}^{\;\mathrm{peaks}}$ is the average EMG peak value calculated.

## Results

Working with EMG data during exercise poses noteworthy levels of inherent noise substantiated through motion artifacts and other phenomena. As a result, when processing data, we identified several recordings that were unusable and were discarded from analysis. The recordings that were excluded exhibited signal saturation, high-levels of noise that obscured contraction identification, general data corruption, or data that exhibited exceptionally high noise floors in the recording that limited measurable contraction peaks. Regardless of this noise, the majority of captured data from over 100 + days of testing were still included in the study. In total, the amount of good hypertrophy data usable for analysis included 281 data points (81.2%), while the bad data points totaled 65 (18.8%). The amount of good endurance data used for analysis included 311 data points (74.9%), while the bad data points were 104 (25.1%). Similarly, the good control hypertrophy data included 22 data points (91.7%), while 2 (8.3%) were unusable. Finally, the control endurance measurements included 7 (87.5%) good data points, while there was only one unusable data point (12.5%). As such, despite our relatively small population, we have a relatively large intraparticipant data set that allows us to explore trends longitudinally for each participant.

### EMG amplitude changes with exercise

**Figure 4:**
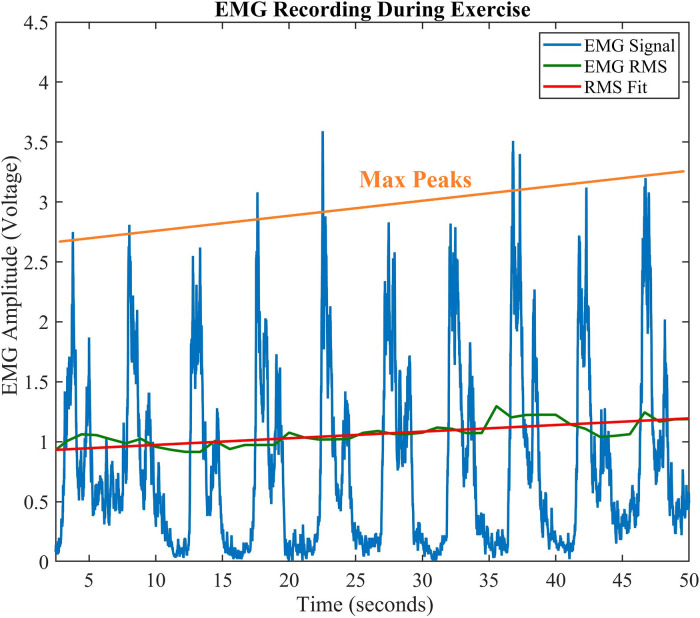
Amplitude vs. Time EMG Signal. Typical progression of EMG during exercise shows an increase in contraction peaks. The orange line shows a regression fitting of the peaks, while the solid green line shows the EMG RMS, and its best linear fit (red line).

A typical rectified EMG signal that is recorded and processed for a single exercise set is shown in Figure 4. The amplitude vs. time plot of the EMG signal shows an increasing trend in the maximum peak amplitude (orange line) and RMS (green/red lines) plots. This is stereotypical across all usable captured data. The peaks correspond to the maximum contraction for that repetition in a set, while the minimum value corresponds to the muscle “relaxing” prior to a repetition. The peak voltage of this rectified EMG signal generally increases from the first to final repetition. In the plot shown, the peak size increases from the first to last contraction by 2.86 V, or 120.74% increase in amplitude. An increase in signal amplitude corresponds with additional motor unit recruitment during a fatiguing task, and varies based on the fatiguing properties of the muscle. We examine the peak change for all exercises below.

### Short term changes in EMG peaks during exercise

**Figure 5:**
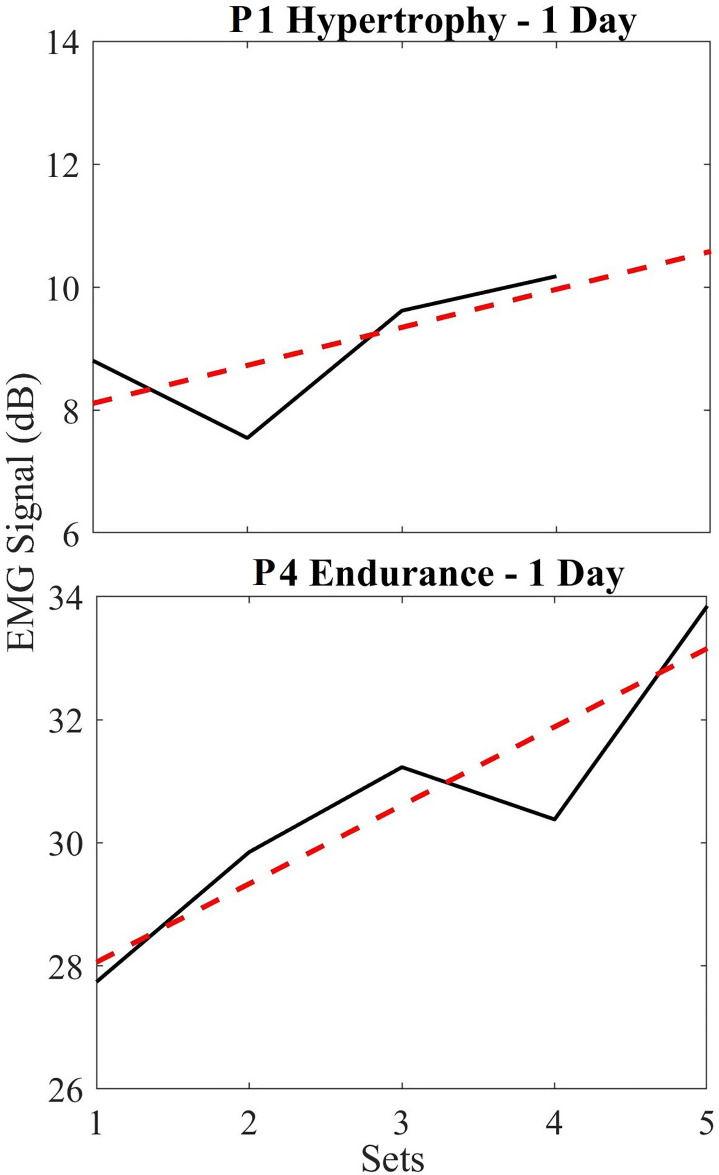
(Top) short term EMG plots for hypertrophy (black) and linear regression trendline (red) for a single day, normalized based on the first set; (bottom) short term EMG plots for endurance (black) and linear regression trendline (red). Notice that these signal sizes are relative for each participant and depend on noise floor, electrode placement, and physiological differences in participants. We are not interested in these values, but rather the progression as shown in the tables and figures below.

Short-term changes in EMG data refer to intraday or intraset behaviors (i.e., on a single day). Figure 5 shows the EMG data using all sets of a day's exercise to depict signal trends over multiple sets. Where, the black lines represent the actual signal amplitude change (first to last contraction) for each set on that particular day and the red line represents the linear fit of these data. For example, in Figure 5 (top), the difference in amplitude from the first contraction to the last contraction was 1.37 dB. Thus, the trend shows a 0.34 dB/set increase across the sets. Similarly, the endurance participant 4 (Figure 5 – bottom) shows an increase of 6.11 dB (1.22 dB/set increase) in EMG signal over the sets performed on the same day. These general trends were found to be typical across all participants, and across all experiments which agrees with the findings in earlier published works (2–5).

We found that this rate of increase in the EMG varied based on when the exercise was performed (i.e., when comparing the first day of their exercise routine vs. day 50). So below, we show this variability in the EMG signal trend by superimposing all normalized short-term endurance and hypertrophy data in two plots (Figure 5). On average, we see a 2.38 ± 3.05 dB increase in EMG signal from endurance participants over 5 sets, while hypertrophy participants show an average of 1.39 ± 3.06 dB increase in EMG signal across 4 sets. These data are normalized such that 0 dB indicates the baseline of EMG (first set) used to compare across sets.

### Long-term changes in EMG during regular exercise

The long-term relative trends in the signal amplitude of the EMG are shown below to identify interday changes (i.e., over the 100 day study period) in muscle performance during regular exercise.

#### Hypertrophy based exercise

**Figure 6:**
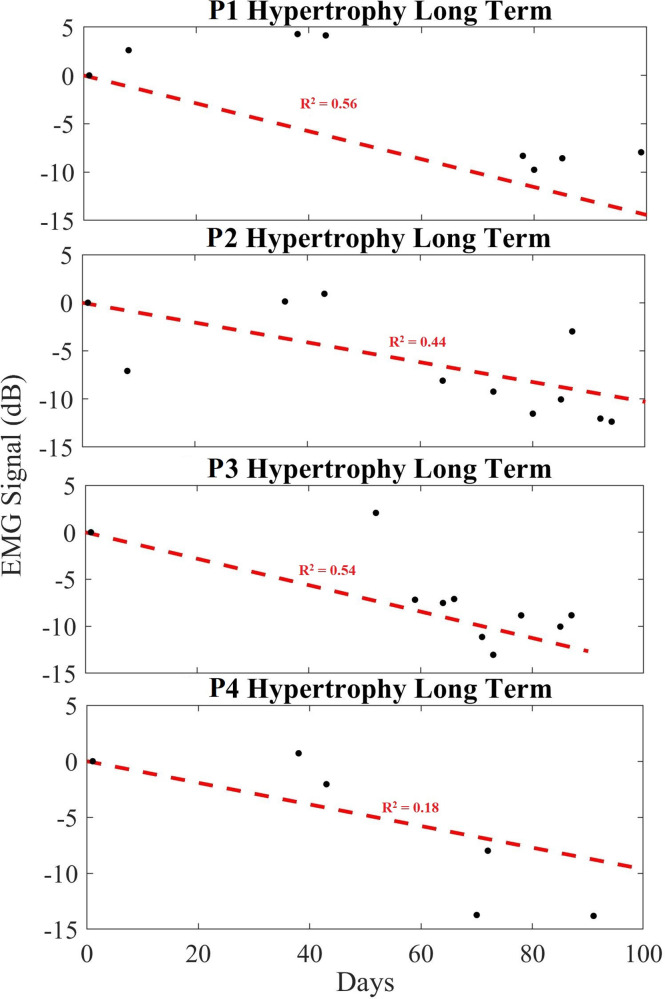
Long-term trend of hypertrophy data showing averaged EMG signal in dB (black), and a linear regression trendline (red) for hypertrophy participants 1 to 4 (P1–P4). Here, we show how the contraction ratio changes over 100 days across sets, rather than for a single day's exercise as per Figure 5. Each black dot indicates the averaged EMG peak data from the sets in dB.

Figure 6 demonstrates the averaged amplitudes of each day's exercise EMG data (each of the 4 sets) for all participants, over a period up to 100 days, performing hypertrophy-based exercises. Recall that data are normalized for each recording using its unique noise floor, and then subsequently normalized based on the day 1 baseline EMG amplitude ratio. The plot shows the data points (black dots) which is the average power in dB of each session consisting of 4 datasets. The “Day 1” data point in the plot corresponds to the first day of data collection during the exercise routine for every participant and last data point in the plot corresponds to the last day of the exercise routine. All data points are plotted based on the relative day the data were obtained. A linear regression (red dashed line) is plotted across the data points to obtain a modeled slope value across the exercise routine completed by each participant. Table 1 indicates the averaged change in EMG signal over the period per month, as well as the total change in relative EMG peak height, and rate change of the EMG as a slope in decibels per day. Here, it is seen that the signal changes across all hypertrophy participants (average of −8.23 dB) indicating a decreasing trend for the long-term. It is important to note the averaged slope of these data is −0.087 dB/day. Participants 1,2 and 4 show an increase in EMG peak amplitude approximately 1 month following the start of the exercise, but the long-term trend persistently decreased.

**Table 1:** Averaged per month EMG signal – hypertrophy.

Averaged EMG Signal (dB)				Signal change (dB)	Avg. Change (dB/day)
Month	1	2	3		
Participants					
1	0	4.72	−8.25	−8.25	−0.083
2	0	4.03	−7.69	−7.69	−0.079
3	0	–	−7.71	−7.71	−0.086
4	0	2.33	−9.26	−9.26	−0.101
			**Average**	**−8.23 **±** 0.63**	**−0.087 **±** 0.008**

#### Endurance exercise

**Figure 7:**
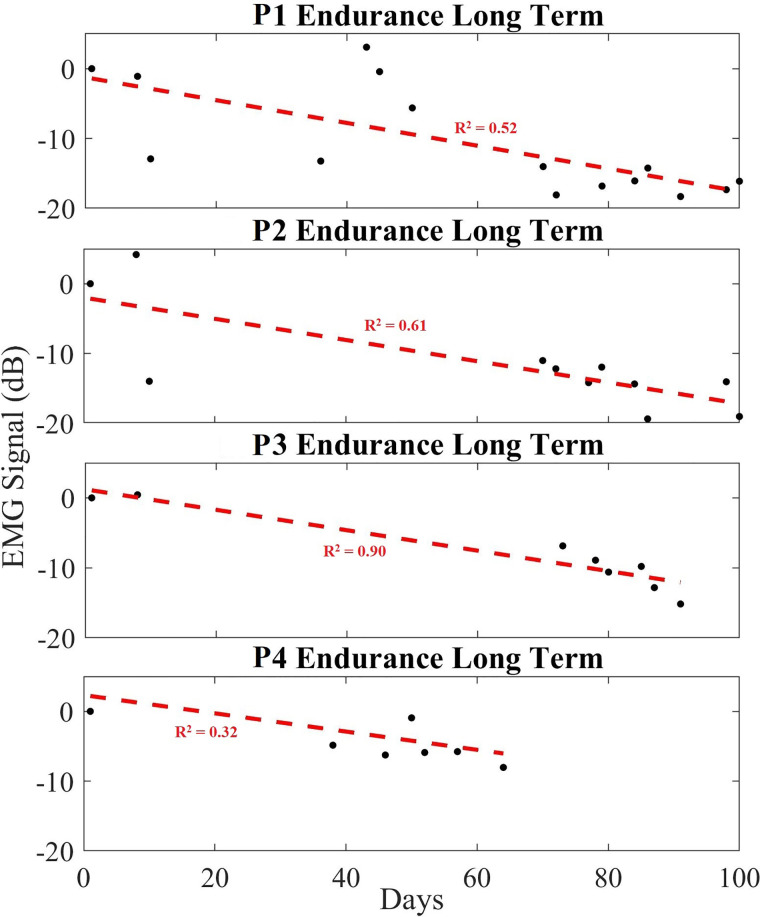
Long-term trend of endurance data showing averaged EMG signal in dB (black), and a linear regression trendline (red) for endurance participants 1 to 4 (P1–P4). Each black dot indicates the averaged EMG peak data from a set in dB.

Figure 7 exhibits the average power of each day's EMG data, over a period of up to 100 days for endurance exercises. The EMG signal amplitude is calculated using all five datasets of each day and plotted over a period of time. The measured data for each recording (black dots) is provided in dB, while a linear regression (red dashed line) is provided to show the trend line. Similar to the data shown in the hypertrophy set, the plotted data show a decreasing trend in the long-term EMG contraction peak size relative to noise floor across all participants. Table 2 shows the averaged change in EMG signal over the exercise period, providing a slope in decibels per day. Namely all participants showed an expected decreasing trend (averaged to −10.09 dB) over the exercise routine period, or −0.113 dB/day in the peak EMG contraction size during regular exercise.

**Table 2:** Averaged per month EMG signal – endurance.

Averaged EMG Signal (dB)				Signal change (dB)	Avg. Change (dB/day)
Month	1	2	3		
Participants					
1	0	−6.18	−14.43	−14.43	−0.144
2	0	−9.38	−11.48	−11.48	−0.115
3	0	–	−8.22	−8.22	−0.093
4	0	−6.21	–	−6.21	−0.100
			**Average**	**−10.09 **±** 3.12**	**−0.113 **±** 0.02**

The data presented for endurance participant #4 contained limited Day 1 and late-stage exercise data due to signal noise, culminating in a reduced Day 1 signal amplitude. Regardless, the stereotypical decreasing trend in EMG contraction peak amplitude was observed.

#### Control exercises

**Figure 8:**
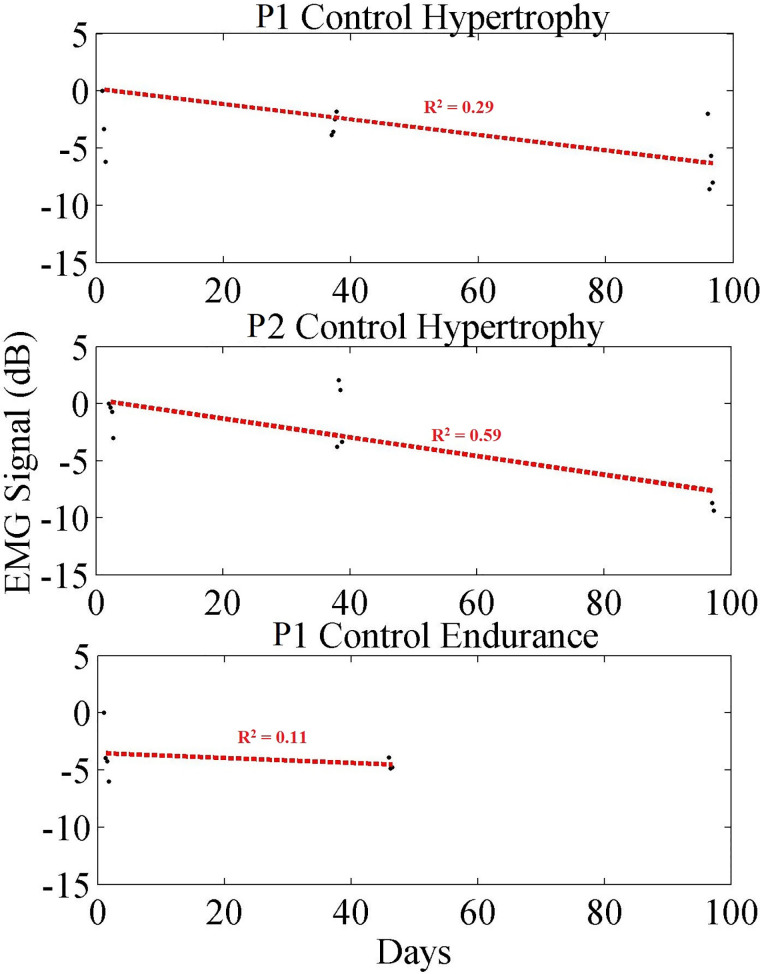
Long-term trend of control participant data showing averaged EMG signal in dB (black) for each exercise set, and a linear regression trendline (red). Each black dot indicates the averaged EMG peak data from a set in dB.

The exercise data for the control participants are plotted in Figure 8. It is shown that for both control groups there are significant differences in outcomes when comparing to their respective exercise groups. Data points collected from each group are averaged and plotted (black dots), while a linear regression (red dashed line) is provided to show the trend. Table 3 summarizes those data, showing the trends for the two hypertrophy control participants and the endurance control participant. Where, the average change in EMG contraction peak size vs. noise floor was −1.57 dB (−0.017 dB/day) and −0.95 dB (−0.020 dB/day), for the Hypertrophy and Endurance respectively. When comparing the control cases to their paired exercise groups, the Hypertrophy group decreased by 6.66 dB (0.067 dB/day) more than the control group. Whilst the Endurance group saw a 9.14 dB (0.093 dB/day) greater reduction than the control group.

**Table 3:** Averaged EMG signal - control based exercise.

Averaged EMG Signal (dB)				Signal change (dB)	Avg. Change (dB/day)
Month	1	2	3		
Participants					
1 (Hypertrophy)	0	−1.99	−3.56	−3.56	−0.038
2 (Hypertrophy)	0	+0.31	–	+0.31	+0.004
			**Average**	**−1.57 **±** 1.99**	**−0.017 **±** 0.021**
3 (Endurance)	0	−0.95	–	−0.95	−0.020
			**Average**	**−0.95**	**−0**.**020**

### Combined data trends

**Figure 9:**
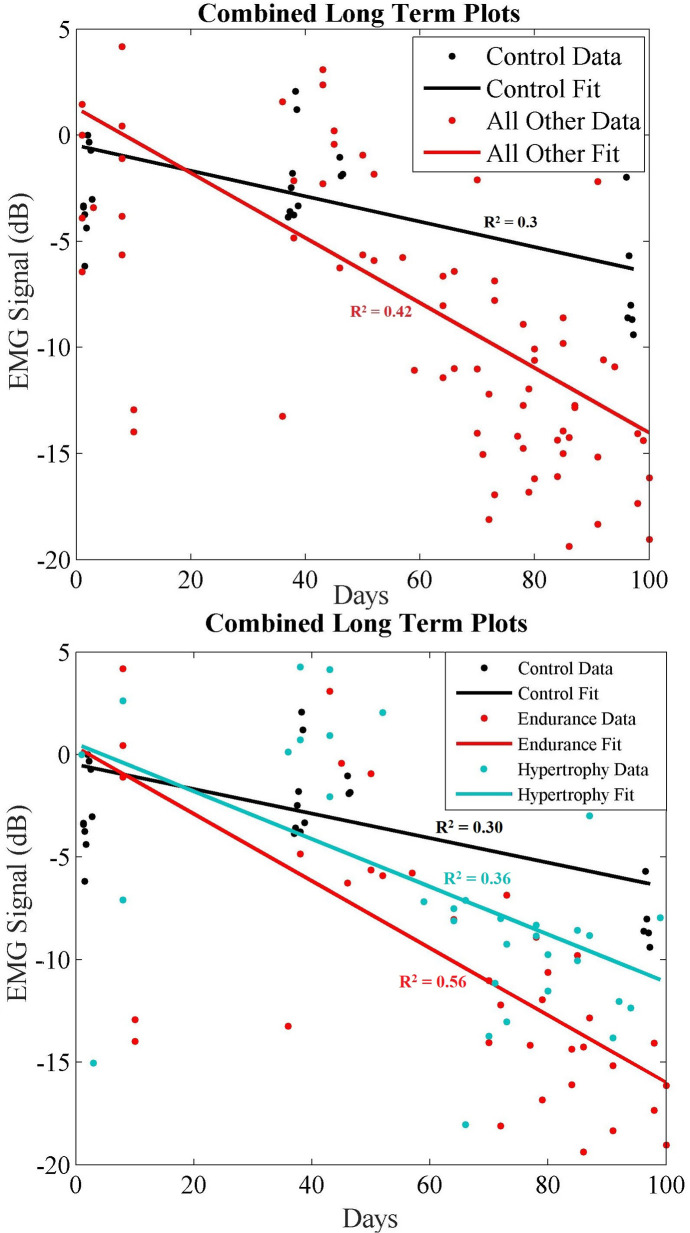
(Top) long-term combined data showing control (black) and all other recorded data (red) where endurance and hypertrophy are grouped together. (Bottom) Long-term combined data showing control (black), endurance (red) and hypertrophy (blue) trends over ∼100 days.

Subsequently, we combined all exercise data into a single plot to highlight the separability of these data. Due to the small population size, we first plot the combined data in two groups (Figure 9 top): (1) the control group; (2) all other participants. Then we plot the same combined data using all groups (i.e., control, endurance and hypertrophy) in Figure 9 (bottom), and we fit regression lines for each group. A comparison of these regression lines is shown in Table 4, where good fits are highlighted in green, and poor fits are red. Namely, we used the correlation of determination between each group's set of data and each regression line. In each case, the R^2^ was best between a group's data and regression. Moreover, the control data did not score well with either endurance and hypertrophy data sets, while the endurance and hypertrophy data showed some correlation.

**Table 4:** Comparing regression lines for each group.

	*R*^2^ Between Regressions			
	C Fit	E Fit	H Fit	Variance
Control	0.30	−1.11	−0.03	16.1
Endurance	−0.19	0.56	0.35	50.9
Hypertrophy	0.13	0.06	0.36	39.0
MMSEs Between Fits
Control	2.3	4.7	3.0	
Endurance	6.4	3.3	4.4	
Hypertrophy	4.7	4.6	3.4	

### Correlation of the time series data

To further explore the separability of these data we examined the time progression of the exercise data with respect to other groups as shown in Figures 6–9. As shown in Table 5, correlations between endurance and hypertrophy is significant (R: 0.71, *p*: 0.000089) while any correlations with the control data set are weak. This must be interpreted with some caution, however, since our study population is small.

**Table 5:** Correlation of long-term data trends.

Correlation of Time Series Data			
	*R* – correlation coefficient		
	Control	Endurance	Hypertrophy
Control		0.03	−0.02
Endurance	0.03		0.71
Hypertrophy	−0.02	0.71	
	*p* - value		
	Control	Endurance	Hypertrophy
Control		0.89	0.93
Endurance	0.89		8.9 × 10^−5^
Hypertrophy	0.93	8.9 × 10^−5^	

## Discussion

This study demonstrates that long-term changes in EMG with regular exercise can be used to quantify the training progression of an individual. Specifically, the rate of change in the EMG peak amplitude relative to a participant's initial baseline can be used to measure adaptation over time. Notably, these data also suggest that the rates of this change are also different depending on the exercise modality (endurance vs. hypertrophy) – however this must be studied further since our study population is small. Finally, we demonstrated that when participants do not undergo regular exercise (control participants), the same long-term changes are not evident due to limited or negligible muscle adaptation. It is evident in Figure 5 that our EMG amplitude data increase across sets within a daily exercise routine. Our data thus confirms what is supported by the literature in short term observations, and coincides with expectations that EMG amplitude increases with muscle fatigue due to the recruitment of additional motor units. This recruitment is necessary to maintain tension required by the muscle when undergoing exercise.

Over a longer period of time, however, as each individual performed resistance-based exercise over their 100 + day study period, the maximum EMG peaks and averaged peak size decreased relative to their Day 1 baseline. It implies that the effort required to do the same task decreased over-time for each individual. This therefore suggests an increase in anti-fatiguing response or other muscle adaptation. For example, each study participant saw their rate of fatigue decrease from Day 1 to Day ∼100 (Tables 1–3). It is worthwhile to observe that the slope of the linear fit of this EMG peak change for each participant is negative. In other words, as the study participants performed exercise over the study period, the EMG saw a slower rate of increase during the fatiguing response. This suggests that the muscles did not recruit as many motor units on the last day of exercise as they did on the first day. Thus, we show that the slope of the long-term trend can be used to indicate the rate at which an individual's muscle becomes more fatigue resistant with regular training.

What is more, when comparing the hypertrophy and endurance participants, their rates were different which also supports our initial hypotheses. For example, when comparing the two groups, the rate of change for hypertrophy and endurance were −0.087 dB/day and −0.113 dB/day, respectively (Tables 1, 2). This means that their slope is different, indicating that the endurance participants saw a greater reduction in EMG peak contraction amplitude during exercise over time relative to the resting EMG amplitude. In this case, it appears that those performing the endurance-based exercise become more fatigue resistant as their rate of motor unit recruitment reduced more over time. This corresponds to what would be expected from an endurance resistance-based exercise. Notice that both hypertrophy and endurance groups exhibit variability over time which can be due to various conditions such as electrode placement changes, physiological differences in participants over time, etc. Similarly, it should be noted that the calculated rates are not predictive across all humans, since the adaptability will be dependent on an individual's unique physiology. Regardless, the downward trend in consistent across all participants over time and the steepness of the slopes seem to be related to the exercise modality.

When comparing these data to the control cases, it shows that these trends are dependent on the exercise. For example, the trends that are evident in Figures 6, 7 are not present in the control participants (Figure 8) since they rarely participated in resistance training.

The exact differences and trends of a wider population will be explored as part of future work that includes work to identify muscle performance changes in neurorehabilitation. These trends in healthy participants provide an important observation toward that goal since we demonstrated that the specific training strategy changes the effect of neuromuscular adaptation, but more importantly that it can be measured in a time-progressing manner. A novel finding in this study demonstrates that the time-constant of the slope of this curve represents an individual's unique change in neuromuscular adaptation over time. We believe that this muscle adaptation time-constant can be an important factor in identifying or predicting training outcomes and identifying an individual's unique training response over time.

Our future studies will explore these effects as part of rehabilitation protocols to improve the quantification of performance and to decrease the rate of fatigue related to training. Based on these findings, we predict that based on an individual's baseline and desired outcomes, specific performance goals can be quantified by using this method. Specifically, the long-term rate of change measures the anti-fatiguing/adaptation response through regular muscle training, which can be measured over time. This potential use includes identification of training interventions when a participant has reached a performance plateau, or has negligible response due to a training plan. This is especially valid for applications in training where fatigue is an overarching limitation. Thus, we suspect that by measuring how an individual responds to their training strategy, modifications to the plan can be made to optimize muscle adaptation and performance (e.g., scaling difficulty, adjusting frequency of exercise etc.) as shown above.

This study has some limitations that we would like to identify. Primarily, we acknowledge the small sample size of this study which includes 8 participants and 3 control participants. Due to the length of the study, particularly in requiring inactive individuals to participate in regular exercise for over 3 months, it proved difficult to recruit a larger cohort. As a result, we would like to clarify we do not claim to model generic inter-participant (i.e., population wide) performance adaptation based on long-term training. Instead, we believe – due to our relatively large intra-participant data set (hundreds of data points) – that these behaviors highlight an individual's performance adaptation that is unique to their ability and physiology. However, we do postulate that the different rates of adaptation are relative to specific training plans as shown above – hypertrophy vs. endurance – which also makes sense physiologically (31, 40). In that same vein, we also omit an analysis that examines the anti-fatiguing or performance differences between males and females regarding resistance-based exercise. Since we predict the exact EMG adaptation trends (change in EMG slope over time) to be unique to each individual, we believe there will be differences between men and women as well and plan on exploring this in the future. Additionally, in this study we omit a wide-bandwidth frequency domain analysis that can be used for fatigue detection, since we hypothesized that lower frequency time-domain responses can be used in identifying the long-term effects of muscle training. Finally, we would like to state that EMG data often exhibit considerable amounts of noise that may skew data, which can create a difficult data capture environment as we experienced with endurance participant #4. Colloquially, there are likely some errors present in the above data due to variations of electrode placement over 100 days of study.

## Conclusion

In this study we presented longitudinal changes to EMG with regular resistance-based muscle training. Using 11 healthy participants who did not regularly weight train, we collected data over 100 days in an attempt to quantify an individual's resultant neuromuscular adaptation. By using the slope of an individual's relative EMG contraction over time, we demonstrated that the rate of fatigue onset decreases over time. Specifically, over 100 days the average EMG exercise set contraction amplitude decreased on average for hypertrophy and endurance-based exercises by −8.23 dB (−0.083 dB/day) and −10.09 dB (−0.113 dB/day), respectively. To the best of our knowledge, this is the first study that demonstrates the measurement of long-term adaptation in muscular activity with regular exercise. We demonstrated that endurance and hypertrophy-based exercise both follow similar trends in this adaptation, but that the rate of change is different. These rates are also different across participants.

By quantifying this adaptation in the time-domain, we hypothesize that the measured rate can inform training strategies for optimization in muscular outcomes. This includes the time of intervention (i.e., shortened training for a better outcome) and to identify when training strategies need to change (i.e., plateauing adaptation effects as shown in our control group). In addition, we propose that this adaptation metric can be used in muscular training protocols to improve interventions in user-customized treatment strategies (healthy individuals or in rehabilitation protocols). Our future studies will explore these capabilities in post-stroke participants.

## Data Availability

The raw data supporting the conclusions of this article will be made available by the authors, without undue reservation.
